# The Effects of TIME-IN on Emotion Regulation, Externalizing, and Internalizing Problems in Promoting School Readiness

**DOI:** 10.3389/fpsyg.2021.579810

**Published:** 2021-04-27

**Authors:** Henk Weymeis, Karla Van Leeuwen, Caroline Braet

**Affiliations:** ^1^Department of Developmental, Personality, and Social Psychology, Ghent University, Ghent, Belgium; ^2^Parenting and Special Education Research Unit, KU Leuven, Leuven, Belgium

**Keywords:** externalizing problems, internalizing problems, emotion regulation strategies, school readiness, school-wide health care policy

## Abstract

Children’s readiness for school is often threatened by the occurrence of both externalizing and internalizing problems. Previous research has shown that Positive Behavioral Interventions and Supports (PBIS) is particularly effective for fostering children’s behavioral skills and reducing externalizing problems. However, whether PBIS can enhance children’s emotional skills and reduce internalizing problems is less clear. Therefore, TIME-IN was developed, which extends PBIS by also including emotional support systems. It was tested whether TIME-IN was effective for (a) improving emotion regulation and (b) reducing depressive symptoms. Furthermore, it was tentatively explored whether TIME-IN is accompanied by more than natural fluctuations in both children’s externalizing and internalizing problems. The effectiveness of TIME-IN was evaluated in a non-randomized study, in which an intervention group was compared with a matched control group. Both research questions were addressed in a sample consisting of 81 children between 8 and 12 years of age with special educational needs. Questionnaires for teachers (i.e., TRF), children (i.e., FEEL-KJ and CDI), and their parents (i.e., CBCL) were administered at the beginning (T0) and the end of the school year (T1) using multi-informant assessment. Only indicative evidence was found for the hypothesis that TIME-IN improved children’s emotion regulation. Practical implications, strengths, and limitations were discussed.

**Clinical Trial Registration:** This work was retrospectively registered at International Standard Registered Clinical/soCial sTudy Number (ISRCTN) registry ISRCTN54456609 (Weymeis, 2017). Registered 28 March 2017.

## Introduction

### Enhancing School Readiness of Children With Special Educational Needs

In a review of the UNICEF (Britto, 2012), school readiness has been broadly conceptualized as successfully adapting to the school environment, which is facilitated by gaining specific competencies (i.e., skills, abilities, and attitudes). In this regard, the current study specifically focused on the behavioral and emotional dimensions of school readiness (see, e.g., Blair and Raver, 2015). With regard to promoting school readiness, UNICEF recommended paying particular attention to young, vulnerable, and/or disadvantaged children with special educational needs. Special educational needs (SEN) has been defined as “learning difficulties or disabilities that make it harder for them to learn or access education than most children of the same age. These children may need extra or different help from that given to other children of the same age” (Westwood, 2007, p. 1).

In Flemish education with a current rate of 60.09%, the largest group of children with SEN is children with learning problems and/or a mild intellectual disability (Flemish Government, 2017). Some of these children receive schooling in regular education, while other more vulnerable children receive special education which can be referred to as “type basisaanbod” education (i.e., cross-national categories A and B; Organisation for Economic Co-operation and Development, 2005; Flemish Government, 2014). Type basisaanbod includes young people with SEN for whom the common curriculum with reasonable adjustments is (temporarily) not feasible in a school for regular education (see, e.g., Farran and Shonkoff, 2010). Because of their specific needs, emotional and behavioral difficulties can also be related to these problems (Dekker et al., 2002; Morgan et al., 2008), in which cause and effect are still difficult to determine. Consequently, the school system (i.e., both regular and special education) in the Flemish part of Belgium is burdened by the load of SEN, concentrated in special education schools. In the current study, specific attention is given to children with learning problems and/or a mild intellectual disability in special education.

Special educational needs children’s emotional and behavioral problems are typically reflected in two well-known variables: externalizing (EP) and internalizing (IP) problems (see, e.g., Baker et al., 2008). EP have been defined as “overt, disruptive behaviors that often involve the violation of societal norms, the destruction of property, and harm towards others” (Keil and Price, 2006, p. 763), whereas IP have been conceptualized as “problems related to anxiety, fear, shyness, low self-esteem, sadness, and depression” (Ollendick and King, 1994, p. 918). Specific attention is needed for this at-risk group given the EP and IP impeding their further school career and possible long-term reintegration into mainstream education. In this regard, research already demonstrated the direct impact of Positive Behavioral Interventions and Supports (PBIS) and its well-known cognitive-behavioral interventions on SEN children’s EP (Cheney et al., 2008; Lane et al., 2008; Horner et al., 2009; Wills et al., 2010), as well as its possible indirect impact on IP (Hunter et al., 2013). However, given the fact that EP and IP have been shown to be highly comorbid (Wolff and Ollendick, 2006), and IP are often underserved, it has been stated by McIntosh et al. (2014) that PBIS policy developers should also deliberately incorporate emotional learning interventions that have been assumed to have direct impact on IP. Emotional learning interventions that seem to meet this criterion are those that are specifically intended for improving children’s emotion regulation (ER), which has been (a) defined by Gross (1998, p. 224) as “processes by which individuals influence which emotions they have, when they have them, and how they experience and express these emotions,” and (b) shown to be a transdiagnostic mechanism that affects both EP and IP (Aldao et al., 2016). Improving emotion regulation is more specifically reflected in acquiring adaptive emotion regulation strategies to the detriment of maladaptive emotion regulation strategies. Important adaptive ER strategies are emotional awareness, identifying emotions, understanding emotions, modifying negative emotions (e.g., through cognitive reappraisal or problem solving), and accepting negative emotions (Aldao et al., 2010; Berking and Lukas, 2015). Well-known maladaptive ER strategies are avoidance, rumination, and suppression (Aldao et al., 2010). Moreover, emotional learning interventions might have the potential to positively affect children with learning problems and/or mild intellectual disability (Bauminger and Kimhi-Kind, 2008; Mcclure, et al., 2009). Interestingly, the adjustments proposed by McIntosh et al. (2014) are in line with the growing tendency to combine PBIS with social and/or emotional learning programs (SEL; see, e.g., Osher et al., 2010; Bradshaw et al., 2014; Cook et al., 2015); however, (a) the integrated version has hardly been investigated so far and (b) to date, very few SEL programs exclusively focus on children’s emotional learning (Rimm-Kaufman and Hulleman, 2016).

**Figure 1:**
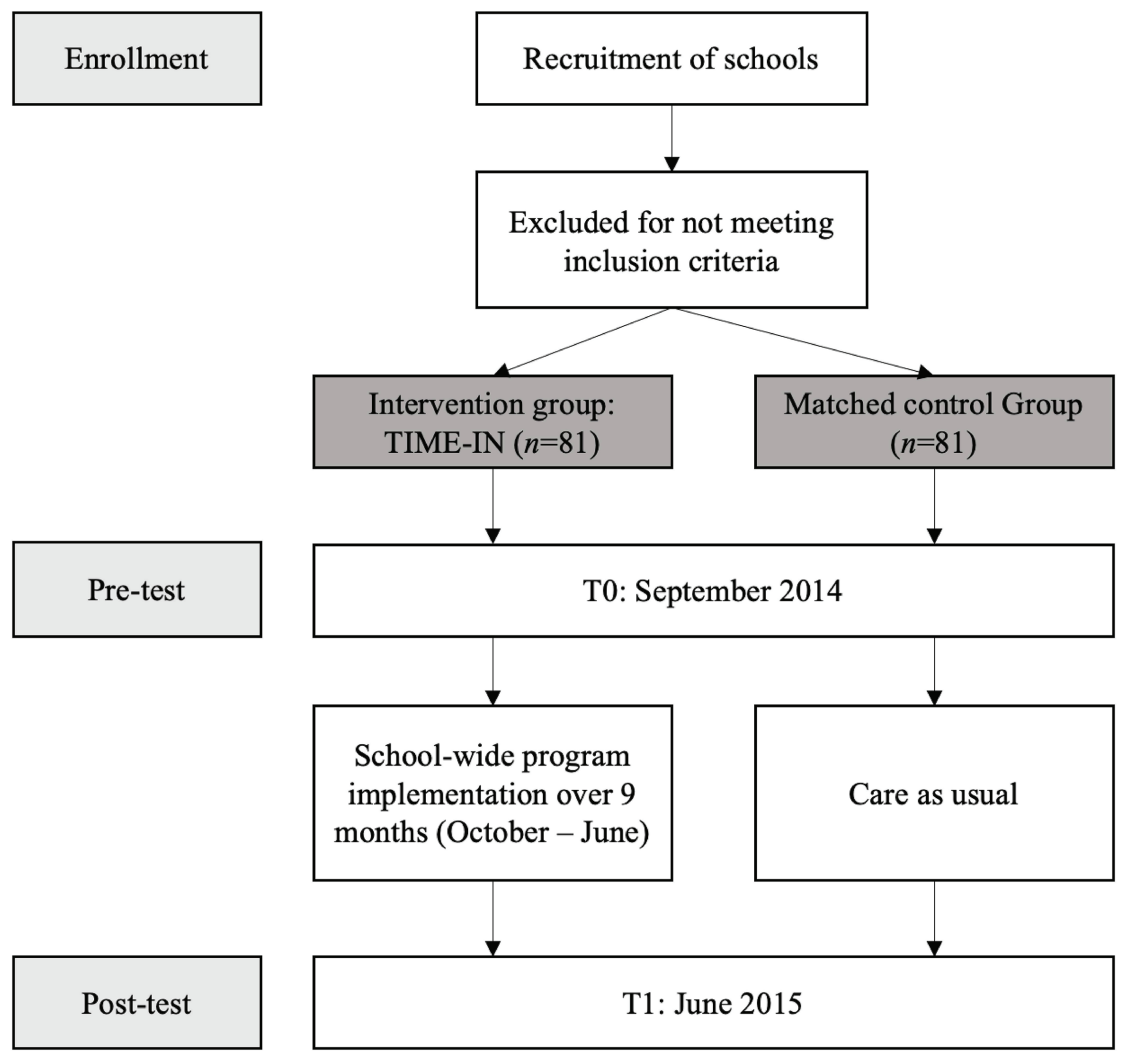
TIME-IN study design. Study progress from enrollment, pre-test to post-test.

To fill this gap, a school-wide health care policy has been developed, named TIME-IN (Weymeis, 2015).^1^ TIME-IN aims to extend PBIS with emotional learning interventions such as (a) screening instruments for identifying IP, (b) emotion regulation training, and (c) crisis intervention strategies. It has been considered useful to train children’s adaptive ER strategies by means of the key principles of Affect Regulation Training (ART; Berking and Schwarz, 2014), which has several advantages since ART integrates different adaptive ER strategies into one coherent model and is effective for reducing various mental health problems (Berking and Lukas, 2015). Currently, ART has only been evaluated in adults and young adolescents (Volkaert et al., 2018), although it was claimed that ART is also applicable to younger age groups (Berking and Schwarz, 2014). Furthermore, to support emotionally overwhelmed children, Life Space Crisis Intervention (LSCI; Long et al., 2003) was used, which was stated to fit well within a school-wide approach (Dawson, 2003), and was found to be effective for children with SEN (Soenen et al., 2014). For a more in-depth description of TIME-IN, including study design and CONSORT diagram (see also Figure 1), visualized continuum of PBIS, program description, and program delivery, see the recently published study protocol (Weymeis et al., 2019b).^2^ The current study will mainly address the question whether TIME-IN is effective for (a) enhancing adaptive ER to the detriment of maladaptive ER and (b) reducing both EP and IP in children with SEN.

### The Current Study: Evaluating TIME-IN

TIME-IN was implemented in a real-life setting (i.e., special education). In the current study, a practice-based evaluation was used to determine whether or not TIME-IN is potentially effective for promoting children’s school readiness (Veerman and van Yperen, 2007; Simons et al., 2016). For an overview of related criteria and considerations, see Weymeis et al. (2019b).

The first goal of the current study was to investigate whether TIME-IN is effective for improving child-reported emotion regulation and decreasing both EP and IP. More specifically, the confirmatory hypotheses were tested that, in the intervention group, TIME-IN was beneficial for enhancing adaptive ER strategies, reducing maladaptive ER strategies, and lowering depressive symptoms. In contrast, it was expected that no such changes would occur in the control group. Furthermore, the second goal of the current study was to tentatively explore whether TIME-IN is also accompanied by reductions in both parent- or teacher reported EP and IP. To deduce whether any reductions in EP and IP are due to the impact of TIME-IN, we controlled for natural fluctuations, related to children’s regular development during this age period (e.g., see Bronfenbrenner, 1977) in a matched control group.

## Materials and Methods

### Participants

The current study was conducted in the Flemish part of Belgium. Participants for both the intervention and control group were recruited using opportunity sampling. One Flemish elementary school providing special education for children between 6 and 12 years of age with SEN was selected by the government as an intervention group. However, it has been stated that well-designed clinical trials consisting of small sample sizes may yield substantial evidence as long as the results are approached in a critical manner (Institute of Medicine, 2001). Participants in the intervention group were 81 Caucasian children between 8 and 12 years of age with SEN (*M*_age_ = 10.27, *SD* = 1.36, 63% boys), as well as their teachers and parents. Unfortunately, insufficient funds were available to prospectively include a control group in the current research project. Therefore, given the opportunity, the control group was retrospectively selected from the concurrent Generation 2020 study (see Van Beveren et al., 2016), which focused on screening children’s school readiness, but did not provide any interventions. More specifically, 11 elementary schools providing regular education for children between 8 and 12 years were included in the study to select the control group. Eighty-one Caucasian children (51 males, mean age = 10.27, *SD* = 1.36), as well as their parents, were randomly recruited from these 11 schools using SPSS case-control matching for age and gender. A full description of the sample is provided in Table 1.

**Table 1:** Sample table for describing participants.

Descriptive variables	Intervention group	Control group
Gender		
Male	*n* = 51 (63.0%)	*n* = 51 (63.0%)
Female	*n* = 30 (37.0%)	*n* = 30 (37.0%)
Total	81	81
Age		
Mean	10.27	10.27
Range	4.0 (8.0–12.0)	4.0 (8.0–12.0)
Race/ethnicity	Caucasian	Caucasian
Socioeconomic status		
Upper class	0.00%	1.20%
Upper-middle class	17.30%	43.20%
Middle class	53.10%	48.10%
Lower-middle class	23.50%	7.40%
Lower class	6.20%	0.00%
Intelligence^*^		
IQ score ≤ 70	*n* = 27 (33.30%)	IQ scores not available
IQ score > 70	*n* = 54 (66.70%)	
Time frame data collection	2005–2014	
Mean	75.61	>90
Range	55–110	
Geographical location	Urban	Urban

* Names of tests used: SON-R, WISC-III, and WPPSI-R.

Regarding the intervention group, children, parents, and teachers who did not give their explicit consent to participate were removed from the study. Furthermore, all children whose age did not correspond to the norm group of the primary measures (6–8 years old; see assessments and measures section) or had a different SEN status (i.e., children with a severe mental health and/or physical disability) were excluded from the study at the time of admission. To be able to accurately identify the capacities of children with learning problems (i.e., IQ score > 70) and/or a mild intellectual disability (i.e., IQ score ≥ 55 and ≤ 70; American Psychiatric Association, 2013), full scale intelligence scores were provided by the school (i.e., secondary data) and derived from three different intelligence tests (Dutch versions): the Snijders-Oomen Non-Verbal Intelligence test-R (SON-R; Laros et al., 1991), the Wechsler Intelligence Scale for Children-III (WISC-III; Kort et al., 2002), and the Wechsler Preschool and Primary Scale of Intelligence-R (WPPSSI-R; Vander Steene and Bos, 1997). These tests were equally standardized (mean = 100.0, *SD* = 15.0) and, moreover, were found to be strongly interrelated (Tellegen et al., 1998). Little’s MCAR test showed no evidence that the data were not missing completely at random for all study variables in the intervention group, *χ*^2^ (58, *N* = 81) = 62.95, *p* = 0.31 (EXT/INT T0: 2.50%; EXT/INT T1: 24.70%; Adaptive/Maladaptive ER T0: 13.60%; Adaptive/Maladaptive ER T1: 11.10%; and CDI T0/T1: 3.70%; Little, 1988). Therefore, missing values were imputed using SPSS expectation maximization (EM). The distribution of scores on the study variables in the normal, subclinical, or clinical range are presented in Table 2.

**Table 2:** The number (*n* =) of children in the normal, subclinical, or clinical range at T0 and T1.

Conditions	T0 = Baseline			T1 = 9 months later		
Intervention group	Normal	Subclinical	Clinical	Normal	Subclinical	Clinical
1. Adaptive ER	72	8	1	71	6	4
2. Maladaptive ER	62	9	10	65	14	2
3. Depressive symptoms	44	25	12	51	17	13
4. EXT	59	11	11	64	11	6
5. INT	58	8	15	66	12	3
Control group	Normal	Subclinical	Clinical	Normal	Subclinical	Clinical
1. Adaptive ER	71	10	0	77	3	1
2. Maladaptive ER	70	10	1	74	6	1
3. Depressive symptoms	66	11	4	64	11	6
4. EXT	70	9	2	73	6	2
5. INT	64	10	7	72	7	2

EXT, externalizing; INT, internalizing; ER, emotion regulation.

With regard to the control group, a full description of the sample is provided in Table 1. Compared to the intervention group, socio-economic status seemed to be differently distributed, *χ*^2^ (4, *N* = 162) = 21.96, *p* < 0.001 in the control group, which included less lower-middle class and more upper-middle class families. Next, IQ scores were not available for the control group, but placement in regular education assumes IQ scores within the normal range (i.e., IQ score ≥ 90; Voeller and Heilman, 1988; Kaufman et al., 2016). Finally, Little’s MCAR test showed that missing data in the control group was missing completely at random, *χ*^2^ (25, *N* = 81) = 31.32, *p* = 0.18 (EXT/INT T0: 2.50%; EXT/INT T1: 45.7%; Adaptive/Maladaptive ER T0: 1.20%; Adaptive/Maladaptive ER T1: 43.20%; CDI T0: 0.0%; and CDI T1: 43.20%). Therefore, missing values were imputed here also using SPSS expectation maximization (EM). The distribution of scores on the study variables in the normal, subclinical, or clinical range are presented in Table 2.

### Procedure

The current study was approved by the ethical committee of Ghent University. Teachers, children, and their parents received a letter consisting of an explanation of the aims and procedures of the study, an invitation to participate, as well as a request to give consent to provide demographics and fill in relevant outcome measures. Consequently, children, parents, and teachers were requested to sign the informed consent form (IC). Also, a short presentation was held in the intervention group to inform parents and teachers about the content of TIME-IN and the related research. Consequently, access was provided to an online tool in order to be able to complete questionnaires at T0 and T1. Children were asked to complete questionnaires on ER and depressive symptoms in both the intervention and the control group. In the intervention group, SEN children received verbal support by repeating items out loud, or by explaining the items in a standardized way using concrete examples. Furthermore, caregivers were requested to complete questionnaires at home or in the classroom. More specifically, teachers were asked to fill out a questionnaire on children’s EP and IP in the intervention group, whereas parents were requested to complete a comparable questionnaire in the control group. The overall data collection was conducted by one additional researcher of Ghent University.

### Assessments and Measures

#### Primary Outcomes

##### Adaptive and Maladaptive ER Strategies: FEEL-KJ

The 90-item Fragebogen zur Erhebung der Emotionsregulation bei Kindern und Jugendlichen was used (FEEL-KJ; Grob and Smolenski, 2005), Dutch version by Braet et al. (2013), to measure a broad range of ER strategies in children and adolescents between 8 and 18 years old. More specifically, the FEEL-KJ assesses different emotion regulation strategies in children’s response to anxiety, sadness, and anger. It obtains two total scores. First, total Adaptive emotion regulation strategies are measured by calculating the scores of seven different strategies: Cognitive Problem-Solving, Problem-Solving, Acceptance, Forgetting, Distraction, Revaluation, and Evoking Positive Mood. Second, total Maladaptive emotion regulation strategies are measured by calculating the scores of five different strategies: Giving Up, Withdrawal, Aggression, Self-Devaluation, and Rumination. Each strategy is measured by rating two items for each of the three emotions, whereby answers are given on a 5-point Likert scale. The FEEL-KJ has been shown to be well-validated and reliable (see Table 3; Cracco et al., 2015).

**Table 3:** Pre- and post-test reliabilities (Cronbach’s *α*).

Study variables	Intervention group T0	Intervention group T1	Control group T0	Control group T1
1. Adaptive ER (FEEL-KJ)	0.91	0.95	0.94	0.96
2. Maladaptive ER (FEEL-KJ)	0.88	0.84	0.82	0.84
3. Depressive symptoms (CDI)	0.78	0.86	0.80	0.89
4. EXT problems (CBCL/TRF)	0.94	0.91	0.85	0.89
5. INT problems (CBCL/TRF)	0.87	0.87	0.82	0.83

ER, emotion regulation; FEEL-KJ, Fragebogen zur Erhebung der Emotionsregulation bei Kindern and Jugendlichen; CDI, children’s depression inventory; EXT, externalizing; INT, internalizing; TRF, teacher report form; CBCL, child behavior checklist.

##### Depressive Symptoms: CDI

The Children’s Depression Inventory (CDI; Kovacs, 1992), Dutch version by Timbremont and Braet (2002), is a 27-item self-report questionnaire for assessing cognitive, affective, and behavioral symptoms of depression in children and adolescents between 7 and 17 years of age. Answers for each item are given on a 3-point Likert scale indicating level of severity. The CDI has been shown to be well-validated and reliable (see Table 3; Smucker et al., 1986; Craighead et al., 1998).

#### Secondary Outcomes

##### Externalizing and Internalizing Problems: TRF and CBCL

The Teacher Report Form (TRF; intervention group) and Child Behavior Checklist (CBCL; control group; Achenbach and Rescorla, 2001), Dutch versions by Verhulst et al. (1996, 1997), respectively, are 113-item questionnaires for measuring teachers’ and parent’s perceptions of 6- to 18-year-old children’s adaptive and maladaptive functioning. The TRF and CBCL are well-validated and reliable (see Table 3; Achenbach et al., 2003). In the intervention study, children’s teachers reported on EP and IP, whereas in the control group, parents were informants of children’s EP and IP. Comparing parents’ and teachers’ reports on these measures seems reasonable, as previous research showed modest cross-informant agreement between parents and teachers regarding children’s EP and IP (Achenbach et al., 2002). However, this is only for descriptive purposes as we are primarily interested in the (experimentally manipulated or naturally) fluctuations between the scores during the 9-month project, thereby comparing pre-test vs. post-test scores of the same informant.

### Data Analytic Plan

Firstly, descriptive statistics, correlations, and the distribution of children in the normal, subclinical, or clinical range were calculated for all outcome variables. Also, the assumption of normality was tested. Secondly, two-tailed independent *t*-tests were performed to check whether the means of the study variables differ significantly between the intervention group and the control group at baseline. Cohen’s effect size (ES) *d* was calculated to determine the size of mean differences (Cohen, 1992). Thirdly, the main study hypotheses were examined by performing separate two-way repeated measures ANCOVA’s for each outcome variable (i.e., Time × Condition). Regarding the first study hypothesis, adaptive ER were controlled for levels of maladaptive ER, while maladaptive ER was controlled for levels of adaptive ER, because adaptive ER and maladaptive ER seem to be correlated (Cracco et al., 2015). Regarding the second study hypothesis, gender differences were controlled for, as it is known from the literature that girls typically experience more IP compared to boys (Crijnen et al., 1997). As EP and IP commonly interfere with each other, EP were controlled for levels of IP, while IP were controlled for levels of EP (Masten et al., 2005). Cohen’s effect size *f* was calculated to determine the interventions’ impact magnitude and the level of significance for all analyses was set at *p* < 0.05. Fourthly and finally, to determine whether there is a clinically significant change in the intervention group for both the primary and the secondary outcome variables, the Reliable Change Index (RCI) was calculated using the formula from Jacobson and Truax (1991). When the RCI is higher than 1.96, the post-test score is likely to reflect a real change.

## Results

### Preliminary Analyses

Descriptive statistics and correlations for all outcome variables are presented in Tables 4 and 5, respectively (Simons et al., 2016). Scores on the primary outcome variables seemed to be positively skewed at T0 and T1 in both the intervention and the control group. Therefore, these were transformed using square root transformation. Furthermore, two-tailed independent *t*-tests showed significant mean differences at baseline between the intervention and the control group for depressive symptoms, *t*(160) = *p* < 0.001, *d* = 0.63 and maladaptive ER, *t*(146.93) = −3.25 = *p* < 0.001, *d* = 0.51. Overall, it can be concluded that the intervention group experiences higher baseline levels of emotional problems compared to the control group.

**Table 4:** Descriptive statistics.

Conditions	T0 = Baseline				T1 = 9 months later			
Intervention group (*n* = 81)	*M*	*SD*	*Min*	*Max*	*M*	*SD*	*Min*	*Max*
1. Adaptive ER	136.01	24.96	72.0	185.0	139.35	29.68	58.0	206.0
2. Maladaptive ER	78.30	18.91	40.0	130.0	74.73	15.76	36.0	118.0
3. Depressive symptoms	12.0	6.24	3.0	30.0	11.38	7.70	2.0	36.0
4. EXT	9.09	10.08	0.0	43.0	7.57	6.94	0.0	37.0
5. INT	7.73	6.82	0.0	27.0	7.69	6.38	0.0	31.0
Control Group (*n* = 81)	*M*	*SD*	*Min*	*Max*	*M*	*SD*	*Min*	*Max*
1. Adaptive ER	140.11	26.92	80.0	195.0	135.60	22.84	81.00	210.0
2. Maladaptive ER	70.81	13.86	36.0	105.0	72.57	12.18	37.11	112.0
3. Depressive symptoms	8.57	5.39	1.0	27.0	9.17	6.24	0.0	34.0
4. EXT	6.29	5.29	0.0	26.0	5.59	5.15	0.0	32.0
5. INT	6.34	5.30	0.0	23.0	5.45	4.19	0.0	22.0

Min, minimum; Max, maximum; EXT, externalizing; INT, internalizing; ER, emotion regulation.

**Table 5:** Correlations (Pearson’s *r*).

Study variables	1	2	3	4	5	6	7	8	9	10
1. Adaptive ER T0	-	0.46^*^	0.14	−0.07	−0.26^**^	−0.07	−0.08	−0.03	−0.002	−0.03
2. Adaptive ER T1		-	−0.16^*^	0.01	−0.14	−0.18^*^	−0.06	−0.001	0.002	−0.06
3. Maladaptive ER T0			-	0.41^**^	0.39^**^	0.39^**^	−0.10	0.04	0.09	0.03
4. Maladaptive ER T1				-	0.26^**^	0.41^**^	0.11	0.20^*^	0.0	−0.07
5. Depressive symptoms T0					-	0.65^**^	0.08	0.15	0.08	0.14
6. Depressive symptoms T1						-	0.13	0.12	0.08	0.09
7. EXT T0							-	0.64^**^	0.37^**^	0.34^**^
8. EXT T1								-	0.15	0.40^**^
9. INT T0									-	0.63^**^
10. INT T1										-

EXT, externalizing; INT, internalizing; ER, emotion regulation.

* *p* < 0.05;

** *p* < 0.01.

### Primary Outcomes

**Figure 2:**
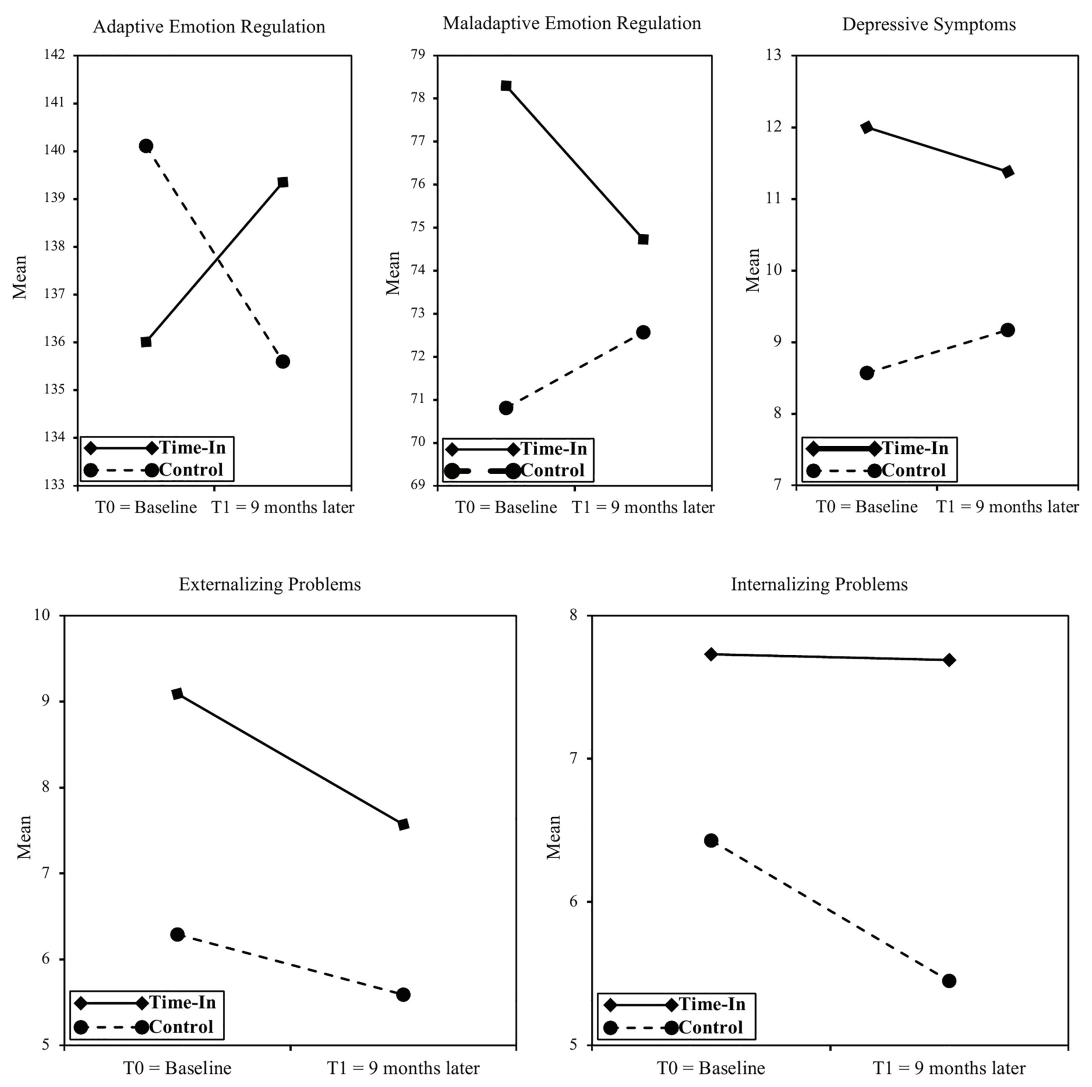
Study variables from baseline to T1 of the intervention and control groups. Only significant Time × Condition effects were found for adaptive and maladaptive emotion regulation.

Regarding the first confirmatory hypothesis, and more concretely children’s adaptive ER, the results showed a significant main effect of time, *F*(1,159) = 18.04, *p* < 0.001 and a Time × Condition interaction *F*(1,159) = 8.28, *p* < 0.01, effect size *f* = 0.23 (see Figure 2), while no significant effect of condition, *F*(1,159) = 0.001, *p* = 0.98 was found. Furthermore, a covariate effect was observed of children’s maladaptive ER, *F*(1,159) = 19.48, *p* < 0.001. Fifteen children in the intervention group (three in the subclinical and 12 in the normal range) showed significant post-test progression (i.e., RCI > 1.96), compared with seven children in the control group (three in the subclinical and four in the normal range).

Regarding children’s maladaptive ER, a significant main effect of time, *F*(1,159) = 7.12, *p* < 0.01, condition, *F*(1,159) = 5.98, *p* < 0.05, and a Time × Condition interaction, *F*(1, 159) = 5.20, *p* < 0.05, effect size *f* = 0.18 (see Figure 2) were found. Furthermore, a covariate effect was observed of children’s adaptive ER, *F*(1,159) = 8.10, *p* < 0.01. Seventeen children in the intervention group (eight in the clinical, four in the subclinical, and five in the normal range) showed significant post-test progression (i.e., RCI > 1.96), compared with eight children in the control group (one in the clinical, four in the subclinical, and three in the normal range).

Finally, concerning children’s depressive symptoms, the results showed a significant effect of condition, *F*(1,157) = 11.31, *p* < 0.001, while no significant main effect of time, *F*(1,157) = 1.86, *p* = 0.18, nor of the Time × Condition interaction, *F*(1,157) = 2.81, *p* = or < 0.10 (see Figure 2) were found. In the intervention group, subclinical and clinical scores for depressive symptoms were found in 46.0% of the children at T0. Nine children in the intervention group (four in the clinical and five in the subclinical range) showed significant post-test progression (i.e., RCI > 1.96), compared with two children in the control group (one in the clinical and one in the normal range).

### Secondary Outcomes

Regarding the second exploratory hypothesis, and more specifically regarding children’s EP, a significant within-subject effect of time, *F*(1,157) = 8.81, *p* < 0.01 was found, while, in contrast, the effect of condition, *F*(1,157) = 0.27, *p* = 0.61, and the Time × Condition interaction, *F*(1,158) = 0.22, *p* = 0.64 (see Figure 2) remained insignificant. Furthermore, a covariate effect was observed of children’s IP, *F*(1,157) = 15.98, *p* < 0.001, while no significant interaction was found between gender and children’s EP. Sixteen children in the intervention group (nine in the clinical, five in the subclinical, and two in the normal range) showed significant post-test progression (i.e., RCI > 1.96), compared with 10 children in the control group (one in the clinical, three in the subclinical, and six in the normal range).

Next, regarding children’s IP, no significant effects of time, *F*(1,157) = 0.31, *p* = 0.58, condition, *F*(1,157) = 1.78, *p* = 0.18, nor Time × Condition interaction, *F*(1,158) = 1.26, *p* = 0.26 (see Figure 2) were found. Fourteen children in the intervention group (eight in the clinical, three in the subclinical, and three in the normal range) showed significant post-test progression (i.e., RCI > −1.96), compared with 19 children in the control group (five in the clinical, nine in the subclinical, and five in the normal range).

## Discussion

The current study evaluated the effectiveness of TIME-IN, a school-wide health care policy for promoting school readiness in children with SEN. As pointed out in the Introduction section and based on the arguments of McIntosh et al. (2014), TIME-IN aims to extend PBIS and its behavioral interventions by providing emotional learning interventions, which are intended for strengthening children’s adaptive ER to the detriment of maladaptive ER, as well as for reducing both EP and IP. More specifically, first, the confirmatory hypotheses were tested whether TIME-IN would be beneficial for improving children’s use of adaptive ER strategies, reducing maladaptive ER strategies and lowering depressive symptoms. Secondly, the exploratory hypotheses were tested whether a reduction of EP and IP would occur in the intervention group and whether the same (natural) fluctuations were observable in a control group.

Regarding the primary study outcomes, the results provided modest evidence that TIME-IN had a positive impact on children’s emotional learning. More specifically, first, there seemed to be a significant increase of adaptive ER strategies in the intervention group. As these improvements mainly concerned children with normal baseline scores, this finding can be an indication of the importance of implementing primary practices for enhancing all children’s emotional competencies (e.g., emotional school and classroom management; see Bradshaw, 2014, p. 99). Secondly, there was also a significant decrease of maladaptive ER strategies. Since this reduction was especially noticeable in children with clinical baseline scores, this result may demonstrate the relevance of implementing secondary and tertiary practices for the most vulnerable children. Thirdly and finally, a trend significant decrease of depressive symptoms (*p* < 0.10) was found, whereby especially children in the subclinical and clinical range showed significant improvements. As the implementation of TIME-IN took place during only one school year, a 9-month intervention period was possibly too short to be fully effective for reducing children’s emotional problems (Litschge et al., 2010). Future studies should, therefore, include a longer intervention period, as well as multiple follow-up measurements.

Regarding the secondary study outcomes, first, an effect of time was observed for all children’s EP, which seemed to imply a natural decrease of children’s behavioral problems over time both in the intervention and control groups. Furthermore, there was no evidence that EP decreased more in the intervention group than in the control group, since time did not seem to interact with condition. Secondly, no significant effects were found regarding children’s IP. We assume that as part of the TIME-IN intervention, teachers in the intervention group became more aware of children’s emotional problems throughout the school year, which might have contaminated the study findings or that external observers (both parents and teachers) were not ideal informants on children’s IP (see, e.g., Theuwis et al., 2013).

As we included only one school for the intervention, the sample included could be too small for strong statistical power and, moreover, this reduced the chance of obtaining reliable and generalizable results (see also Parker, 1990). According to power tables of Cohen (1988, p. 55), however, a total sample size of 28 children per condition should have been sufficient to generate sufficient power (i.e., 1 − *β*). Furthermore, combining a small sample size with a relatively high significance level (i.e., 0.05) increases the probability of accepting a false null hypothesis, leaving potential significant effects undetected (i.e., Type II error). Therefore, the optimal level of significance might have to be set higher than 0.05 (e.g., 0.10; Kim and Choi, 2019).

### Practical Implications

The above results entail different implications for promoting school readiness in children with SEN. Firstly, the beneficial impact of TIME-IN on children’s ER, as well the observed downward trend regarding children’s depressive symptoms, could deliver a rationale for facilitating the overall implementation process of a positive school-wide health care policy on both the schools’ meso and micro level, since this contradicts teachers’ often persistent conviction that disciplinary practices are the most effective way to address children’s behavioral and emotional problems (see Sugai and Horner, 2002; Beets et al., 2008). In addition, as implementation efforts are often accompanied by stress, feelings of incompetence, and resistance to change, these results may convince teachers to participate during sustained implementation efforts (Evers et al., 2002).

Secondly, the results suggest that, besides the implementation of well-known behavioral practices, it is useful for schools to also include emotional learning interventions in special education (McIntosh et al., 2014). More specifically, to enable children to deal with academic stress and related emotions, teachers could be professionalized in screening and training children’s emotional competencies such as adaptive ER strategies (Davis and Levine, 2012).

### Strengths and Limitations

Regarding study strengths, first, reliable measures were used, which decreased possible error variance and, as such, increased the study’s statistical power. Secondly, the current study had the potential to compensate for shortcomings in experimental research as it was conducted in a real-life setting and, as a result, provided “richer” data and increased ecological validity (Schmuckler, 2001). Thirdly, the current study included longitudinal data (i.e., T0 and T1), as well as a matched control group, which may have yielded preliminary signs of causality on the assumed relations between the study variables (Maxwell and Cole, 2007; Veerman and van Yperen, 2007; Simons et al., 2016). This implicated that, within a short time range of 9 months, modest statements could be made about the effects of TIME-IN on children’s use of ER strategies. Fourthly and finally, this was one of the first studies investigating a school-wide intervention that aimed to extend PBIS by adding an emotional learning intervention and, moreover, by specifically focusing on improving children’s emotional readiness.

Regarding study limitations, first, a design issue occurred. More specifically, there was a lack of randomization, which increased the chance that uncontrolled factors were unevenly distributed over the intervention and the control group. Moreover, both conditions were matched regarding demographical characteristics such as gender and age. However, due to the use of opportunity sampling, some baseline scores for the intervention and control groups significantly differed, which suggests that we were not able to take into account maturation effects caused by both child (e.g., SEN status, IQ, and psychosocial problems) or environmental (e.g., SES, educational context, and differences in implementation) factors. Secondly, other threats to internal validity may have occurred, such as testing, regression, differential selection (e.g., bias due to differences between the intervention and control groups related to the composition of normal, subclinical, and clinical scores on the study variables), and selection-maturation interaction (Slack and Draugalis, 2001). All these issues reduced the ability to draw causal conclusions about the effect of TIME-IN on children’s school readiness (Simons et al., 2016). To resolve these issues, and to be able to conclude that TIME-IN was efficacious, evidence is required that the presumed outcomes are caused by the intervention and/or its presumed working mechanisms (Veerman and Van Yperen, 2007). In this regard, causal statements typically arise from rigorous evaluations such as a randomized controlled trial (RCT) and/or a single case study. At this moment, we have not yet been able to carry out such research, but, however, we did manage to conduct an additional study to explore change mechanisms (Weymeis et al., 2019a). Thirdly, another study limitation is related to the single use of questionnaires, which may have resulted in shared method variance. This issue could, however, be addressed in the future by including other data sources such as observations, interviews, and children’s concrete test results. Fourthly and finally, some issues may have occurred due to the use of teacher- and/or parent reports for our secondary outcome measures. Scores for EP and IP were obtained by different informants in the intervention and control groups (teachers and parents, respectively), which could have led to distorted or tentative results (Simons et al., 2016), as it complicates a reliable comparison. As we were interested in the (experimentally manipulated or naturally) fluctuations between the scores during the 9-month project thereby comparing pre-test vs. post-test scores of the same informant, we believe that the findings on IP and EP were informative to include.

### Conclusion

The current intervention study investigated whether TIME-IN, which extends PBIS by adding an emotional learning intervention, was beneficial for fostering children’s school readiness. Overall, the results provided indicative evidence that TIME-IN improved children’s ER, which, as a result, may convince schools and teachers to also sustainably implement emotional practices as a classroom management strategy.

### Resource Identification Initiative

This work was retrospectively registered at International Standard Registered Clinical/soCial sTudy Number (ISRCTN) registry ISRCTN54456609 (Weymeis, 2017).^3^ Registered 28 March 2017.

## Author’s Note

The authors partially reused T1 data from American Psychological Association (2011) and Weymeis et al. (2019a). The authors furthermore assure that the findings contribute to knowledge/practice relative to what has already been published in Weymeis et al. (2019a), in accordance with the data reuse guidelines formulated by van Raaij (2018).

## Data Availability Statement

The raw data supporting the conclusions of this article will be made available by the authors, without undue reservation.

## Ethics Statement

The studies involving human participants were reviewed and approved by Ethical Committee of Ghent University Henri Dunantlaan 2, 9000, Ghent Belgium https://www.ugent.be/pp/nl/onderzoek/ec. Written informed consent to participate in this study was provided by the participants’ legal guardian/next of kin.

## Author Contributions

HW wrote this report on this TIME-IN clinical trial, assisted with feedback provided by CB (promotor) and KL (co-promotor). All authors read and approved the final manuscript.

### Conflict of Interest

Braet et al. (2013) translated the FEEL-KJ (Grob and Smolenski, 2005) and received an authorship fee for the official version.

The remaining authors declare that the research was conducted in the absence of any commercial or financial relationships that could be construed as a potential conflict of interest.

## Footnotes

^1^https://www.acco.be/nl-be/items/9789462922884/Wij-zijn-gedrag

^2^http://journals.ed.ac.uk/social-science-protocols/article/view/3038 (public access).

^3^http://www.isrctn.com/ISRCTN54456609?q=&filters=&sort=&offset=3&totalResults=15635&page=1&pageSize=100&searchType=basic-search

## Supplementary Material

To ensure complete transparency, a data transparency matrix was submitted as supplementary material (American Psychological Association, 2011).
