# Supplementary material for: The Effects of TIME-IN on Emotion Regulation, Externalizing, and Internalizing Problems in Promoting School Readiness
Source: Front Psychol. 2021 Apr 27;12:579810. doi: 10.3389/fpsyg.2021.579810 (PMC8111215; doi:10.3389/fpsyg.2021.579810)
Supplement: Supplementary file 1 [file Table_1.pdf]

**Variables in the Complete Dataset**[1]	**MS 1**[2] **(STATUS=pub)**	**MS 2 (STATUS=current)**
Externalizing problems (CBCL) T0		x
Externalizing problems (CBCL) T1		x
Externalizing problems (TRF) T0	x[3]	x
Externalizing problems (TRF) T1		x
Internalizing problems (CBCL) T0		x
Internalizing problems (CBCL) T1		x
Internalizing problems (TRF) T0	x	x
Internalizing problems (TRF) T1		x
Depressive symptoms (CDI) T0		x
Depressive symptoms (CDI) T1		x
Adaptive emotion regulation (FEEL-KJ-TR) T0	x	x
Adaptive emotion regulation (FEEL-KJ-TR) T1		x
Maladaptive emotion regulation (FEEL-KJ-TR) T0		x
Maladaptive emotion regulation (FEEL-KJ-TR) T1		x

[1] American Psychological Association. (2011). Data Transparency Appendix Examples. Retrieved September 29, 2020, from https://www.apa.org/pubs/journals/apl/data-transparency-appendix-example

[2] Weymeis, H., Van Leeuwen, K., and Braet, C. (2019a). Adaptive emotion regulation, academic performance and internalizing problems in Flemish children with special educational needs: A pilot study. *European Journal of Special Needs Education* 34(1), 124-135. doi: 10.1080/08856257.2017.1421601

[3]Included as control variable, not as study variable
